# Sorting beef subprimals by ribeye area size at the packer level to optimize utility and product uniformity in foodservice and retail

**DOI:** 10.1093/tas/txaa107

**Published:** 2020-07-02

**Authors:** Chandler C Steele, Ashley N Arnold, Kerri B Gehring, Davey B Griffin, Jeffrey W Savell

**Affiliations:** Department of Animal Science, Texas A&M AgriLife Research, Texas A&M University, College Station, TX

**Keywords:** beef, beef carcass, beef subprimals, LM area, sorting

## Abstract

The objectives of the study were to evaluate if sorting beef carcasses at the packer level by loin muscle (LM) area, using instrument grading technology, would increase the consistency of three boxed beef products for the foodservice and retail sectors of the industry. U.S. Department of Agriculture (USDA) Choice beef sides (*n* = 100) and USDA Select sides (*n* = 100) were selected and stratified into five LM area categories (±2.9 cm^2^): 1) 77.4, 2) 83.9, 3) 90.3, 4) 96.8, and 5) 103.2 cm^2^. Beef lip-on ribeyes and boneless strip loins were obtained from USDA Choice sides and full, partially defatted tenderloins were obtained from USDA Select sides. Subprimals were scanned with a portioner that captured visual images and dimensional analyses of each subprimal, and data were analyzed by the software to determine multiple portioning outcomes for each subprimal. Portioning data were generated for each subprimal based on a variety of targeted portion weights (ribeye and strip loin steaks = 340.2 g; tenderloin steak = 170.1 g), as well as various portion thicknesses (ribeye and strip loin steaks = 31.8 mm; tenderloin steak = 44.5 and 50.8 mm). Subprimal utility varied across targeted portion weights and thicknesses within each LM area category. For the ribeyes and strip loins, optimal portion weight and thickness combinations were observed more frequently in LM area categories 1 and 2 than for the three larger LM area categories. Analysis of data for tenderloins revealed that LM area categories played a lesser role in identifying optimization of steak portion weight and thickness combinations. Findings demonstrate that creating categories of beef subprimals based on LM area as opposed to subprimal weight might provide a unique sorting method that would improve boxed beef product consistency and uniformity for foodservice and retail sectors.

## INTRODUCTION

The National Beef Quality Audit (NBQA) is an industry-led, data-driven initiative completed approximately every 5 years to identify trends in the U.S. beef industry. Over the last 20 years, these audits have revealed progressively heavier beef carcass weights (Boleman et al., 1998; McKenna et al., 2002; Garcia et al., 2008; Moore et al., 2012; Boykin et al., 2017), and U.S. Department of Agriculture (USDA), Economic Research Service livestock and meat marketing information (USDA, 2020a, 2020b) confirms that this trend has been occurring over many decades. Today, beef packers and purveyors are challenged to manage the variable supply of beef carcass weights while providing uniform products to downstream segments of the beef supply chain. Due to the variable weights of beef carcasses and subsequent cut sizes entering the foodservice and retail segments of the industry, new strategies are needed to ensure that the value chain is supplied with consistent products that promote a favorable eating experience for the consumer.

Currently, at the packer level, several beef subprimals are sorted by weight during boxing into “ups” and “downs” based on the box containing products above or below a set weight threshold, respectively. Sorting subprimals by weight in this manner only minimally provides foodservice operators and retailers with uniform products due to the opportunity for size variability that may remain within and between boxes. Improving the consistency and uniformity of beef products purchased by these segments of the industry may lead to more useful utilization of products and improve the ability to provide consumers with a quality eating experience.

McKenna et al. (2016) showed success in portioning strip loins to a targeted portion weight while maintaining acceptable cut thickness by selecting subprimals of a specific loin muscle (LM) area when compared to an unsorted control group. Today, all major beef packers have implemented instrument grading technology to efficiently and effectively measure carcass characteristics, including LM area. Existing instrument technology could be utilized to segment carcasses based on LM area rather than sorting individual subprimals by weight. The current study sorted beef carcass sides into five LM area categories before the carcass sides were fabricated into ribeyes, strip loins, and tenderloins, which were subsequently assessed for cutability into a variety of portions based on thickness and weight. Use of technology to assess both LM area through instrument grading and portioning options through state-of-the-art computer-assisted portioning systems allowed the opportunity to obtain data that have only existed in recent time.

## MATERIALS AND METHODS

### Carcass Selection

Beef sides were selected from a commercial beef harvest and processing facility. Instrument grading technology was utilized to identify 100 USDA (2017) Choice, yield grade 2 and 3 sides and 100 USDA (2017) Select, yield grade 2 and 3 sides that were further sorted into one of five LM area categories (*n* = 20 carcass sides per category; Table 1). Selected sides were tagged, and the individual carcass side identification numbers provided by the packing establishment were recorded. Carcass quality and yield factors were obtained from the instrument grading system and are reported in Table 2.

**Table 1.** LM area categories and associated acceptable LM area ranges

LM area category	LM area, cm^2^	Allowable range, cm^2^
1	77.4	74.8–80.6
2	83.9	81.3–87.1
3	90.3	87.7–93.5
4	96.8	94.2–100.0
5	103.2	100.6–106.4

**Table 2.** Mean carcass attributes

	Choice (*n* = 100)	SD	Select (*n* = 100)	SD	Combined (*n* = 200)	SD
Hot carcass weight, kg	357.7	32.8	364.7	40.2	361.2	36.8
LM area, cm^2^	90.5	9.0	91.2	8.9	90.8	8.9
Yield grade	2.7	0.5	2.6	0.5	2.7	0.5
Marbling score^*a*^	445	27	367	20	406	46

^*a*^Marbling score: 300 = Slight^00^; 400 = Small^00^; 500 = Modest^00^ (USDA, 2017).

### Carcass Fabrication

All carcass sides were fabricated to produce subprimals that complied with the Institutional Meat Purchase Specifications (IMPS), as described by USDA (2014) and the North American Meat Institute (2014). Beef rib, ribeye, lip-on (IMPS 112A) and beef loin, strip loin, boneless (IMPS 180) were obtained from each USDA Choice side identified for the study. Additionally, a beef loin, tenderloin, full, side muscle on, partially defatted (IMPS 189B) was removed from each USDA Select side identified for the study. All subprimals were individually vacuum packaged, boxed, shipped using a refrigerated truck (0 °C) to a commercial foodservice operator, and held under refrigerated storage (−0.5 °C). A few subprimals lost their identification in the fabrication process resulting in 95 tenderloins, 98 strip loins, and 97 ribeyes available for the remaining phases of the study.

### Subprimal Fabrication

After the completion of a 15-d aging period, tenderloins (*n* = 95) were unboxed, organized by individual identification number, unbagged, and weighed using two Marel M Series 1100 Scales (Marel, Lenexa, KS) to yield pretrimmed subprimal weights. Establishment employees removed the side muscle (M. psoas minor) and the principal membranous tissue covering the M. psoas major, converting them to beef loin, tenderloin, full, side muscle off, skinned (IMPS 190A). Each trimmed subprimal was reweighed.

After the completion of a 27-d aging period, strip loins (*n* = 98) and ribeyes (*n* = 97) were unboxed, unbagged, and weighed using an Adam Equipment WBW 18a, max 8 kg (Adam Equipment, Inc., Oxford, CT) and a Gainco Infiniti Digital scale, max 8 kg (Gainco, Inc., Gainesville, GA). Establishment employees prepared each strip loin by trimming external fat to not exceed 7 mm over the dorsal surface, removing remnant lean and fat tissues on the ventral side flush with the lean surface and ensuring that the flank edge did not exceed 25 mm on the rib end and 0 mm on the sirloin end from the M. longissimus lumborum. The ribeyes had the “lip” (M. serratus dorsalis and M. longissimus costarum and related intermuscular fat) on the short plate side removed along the natural seam immediately lateral to the M. longissimus thoracis, as well as any surface fat, M. intercostales interni and M. intercostales externi remaining on the ventral side to produce beef rib, ribeye roll (IMPS 112). Trimmed strip loins and ribeye rolls were weighed again.

To assess cutting options, each subprimal was passed through a Marel Portioner—M Series 3000 (Marel, Lenexa, KS) to obtain a visual image and dimensional analysis. Scan data were used by Marel IPM3 Simulation software (Marel, Lenexa, KS) to calculate optimal or desired cut configurations, allowing the determination of multiple portioning outcomes for each subprimal. The software program predicted the yields of steaks and lean trimmings (e.g., facings and end cuts) for each subprimal when the portion simulation was performed based on 1) portion weights or on 2) portion thicknesses. The steak endpoints were beef loin, tenderloin steak, side muscle off, skinned (IMPS 1190A); beef loin, strip loin steak, boneless (IMPS 1180); and beef rib, ribeye roll steak, boneless (IMPS 1112).

### Statistical Analyses

Data were analyzed using JMP Pro software (version 14.0, SAS Institute Inc., Cary, NC). Data were generated and reported by portion weight by LM area category. Data then were analyzed by portion thickness by LM area category using the same methods. Qualitative assessments were made of the appropriateness of each portion and method within and across the LM area categories. The Fit Y by X function was used for ANOVA, and least-squares means comparisons were conducted using All Pairs, Tukey HSD. Correlation coefficients between hot carcass weight and LM area were determined using the multivariate function. Mean values were determined using the distribution function.

## RESULTS AND DISCUSSION

### Carcass Information and Subprimal Weights

Mean carcass attributes for the USDA Choice, USDA Select, and combined quality grades are shown in Table 2. The combined mean hot carcass weight was 361.2 kg with USDA Choice carcasses being lighter numerically and USDA Select carcasses being heavier numerically. The average hot carcass weight for this study was lighter than the average hot carcasses weight (390.3 kg) reported in the NBQA-2016 inplant survey (Boykin et al., 2017). This finding could be a result of the defined LM area categories, which limited the number of heavier- and lighter-weight carcasses from being included in this study. The NBQA-2016, in-plant survey (Boykin et al., 2017), revealed a positive correlation (*r* = 0.40) between carcass weight and LM area. In this investigation, in data not reported in tabular form, the correlation coefficients between carcass weight and LM area was 0.72 for Choice, 0.56 for Select, and 0.63 for the combined grades (*P* < 0.0001 for all coefficients).

Weights of the subprimals before and after trimming, as well as yields, are presented in Table 3. As might be expected, subprimals, whether in their original form or as trimmed, were significantly heavier as the LM area categories increased in size. Yields of trimmed subprimal weights compared to their original weights revealed no differences (*P* > 0.05) among the LM area categories for any of the subprimals. The LM area categories impacted weights of subprimals, but yields were unaffected as subprimals were prepared for eventual steak cutting.

**Table 3.** Least squares means (±SEM) for subprimal weight and trimmed subprimal weight by LM area category^*a*^ for ribeyes, strip loins, and tenderloins

Subprimal/LM area category	Subprimal weight, kg	Trimmed subprimal weight, kg	Yield, %
Ribeye			
1	6.2^c^ ± 0.1	4.5^c^ ± 0.1	72.4 ± 0.6
2	6.5^c^ ± 0.1	4.8^c^ ± 0.1	73.1 ± 0.6
3	7.0^b^ ± 0.1	5.1^b^ ± 0.1	73.1 ± 0.6
4	7.2^b^ ± 0.1	5.3^b^ ± 0.1	73.7 ± 0.6
5	8.0^a^ ± 0.1	5.9^a^ ± 0.1	73.9 ± 0.6
*P*-value	<0.0001	<0.0001	0.3521
Strip loin			
1	5.7^cd^ ± 0.1	4.1^c^ ± 0.1	72.6 ± 0.8
2	5.6^d^ ± 0.1	4.2^c^ ± 0.1	74.2 ± 0.8
3	6.2^bc^ ± 0.1	4.6^b^ ± 0.1	75.1 ± 0.8
4	6.3^b^ ± 0.1	4.6^b^ ± 0.1	72.6 ± 0.8
5	6.8^a^ ± 0.1	5.1^a^ ± 0.1	74.7 ± 0.9
*P*-value	<0.0001	<0.0001	0.1101
Tenderloin			
1	2.9^c^ ± 0.1	1.9^c^ ± 0.1	65.2 ± 0.7
2	3.0^bc^ ± 0.1	2.0^bc^ ± 0.1	64.8 ± 0.7
3	3.1^abc^ ± 0.1	2.1^abc^ ± 0.1	65.4 ± 0.7
4	3.2^ab^ ± 0.1	2.1^ab^ ± 0.1	65.2 ± 0.6
5	3.3^a^ ± 0.1	2.2^a^ ± 0.1	66.2 ± 0.6
*P*-value	<0.0001	<0.0001	0.6663

^*a*^LM area categories: 1 = 74.8–80.6 cm^2^; 2 = 81.3–87.1 cm^2^; 3 = 87.7–93.5 cm^2^; 4 = 94.2–100.0 cm^2^; and 5 = 100.6–106.4 cm^2^.

^a,b,c,d^Means within each column for each subprimal with different superscripts differ (*P* < 0.05).

### Portion Weight/Portion Thickness Outcomes

We sought input from our foodservice collaborator regarding target portion weights and portion thicknesses that would meet most of the requirements for its customers or would have the greatest utility for many restaurant operations. For the ribeye, 12-ounce (340.2 g) portions were deemed the most common weight endpoint with some demand for 10-ounce (283.5 g) portions and 16-ounce (453.6 g) portions. For the strip loin, 12-ounce (340.2 g) portions were the most common portion weight with some limited demand for 14-ounce (396.9 g) portions. For the tenderloin, 6-ounce (170.1 g) portions were deemed the most common with some demand for heavier 8-ounce (226.8 g) and 9-ounce (255.2 g) portions. The target portion weights of 340.2 g for both the ribeye and strip loin steaks and 170.1 g for the tenderloin steak were chosen. Because there are a variety of portion weights that are used in both retail and foodservice, we have provided several options that may fit most merchandising scenarios.

For portion thicknesses, 31.8 mm was targeted as optimal for both ribeye and strip loin steaks and, for tenderloins, both 44.5 and 50.8 mm were deemed as target thicknesses. Portion thicknesses may be a part of customer specifications for companies that provide products for the restaurant industry even though portion weights are what are featured on most menus. For case-ready operations, many use a variety of portion thicknesses, depending on the cut and the market, to produce steaks and roasts for the retail market. Data have been generated for portion thicknesses above and below the target portion sizes so that various combinations of portion thicknesses and portion weights can be evaluated. Using the portion-cutting technology for this study provided the opportunity to simulate multiple endpoints to assess the role of smaller to larger LM areas in achieving optimum steak portions for a particular marketplace.

Past work on optimum steak size and thickness is conflicted. Leick et al. (2011) found that consumers ranked steak thickness as the first or second most important selection criteria for ribeye, top loin, and top sirloin steaks. However, at least for ribeye steaks, when all were portioned to the same weight, although from different LM area/carcass weight combinations, consumers preferred steaks from the largest LM area, possibly due to the greater surface area. This greater surface area as preference/selection criteria was mentioned by both Leick et al. (2012) and Sweeter et al. (2005). Leick et al. (2011) stated that there are challenges in setting thickness/size parameters because of differences in demographics, which helps support our use of multiple portion thickness/portion weight combinations. Conversely, in an economic study designed to evaluate the consequences of larger/heavier beef in the marketplace, Maples et al. (2018) found that consumers tended to value thickness slightly more than surface area and that the majority of steak consumers disliked thin-cut steaks. Finally, Dunn et al. (2000), in a study where steaks from different LM area categories were cooked and evaluated, found that those from LM areas between 77.4 and 96.6 cm^2^ had optimal tenderness and cooking times, features benefiting foodservice applications.

The portion-cutting software generated a variety of datapoints that were used to assemble the information reported for ribeyes, if portioned by weight (Table 4) or by thickness (Table 5), when stratified by LM area category. For the ribeyes portioned by weight, steaks from LM area categories 1 and 2 had steaks that approached or met the 31.8-mm thickness target for two weights: 340.2 and 396.9 g. This would be especially important for the 340.2-g category because that would be a steak that met both the portion thickness and portion weight targets. For the remaining LM area categories (3, 4, and 5), only those steaks that weighed 396.9 g approached or met the thickness target of 31.8 mm. In fact, for LM area category 5, steaks lacked 1 mm from meeting the target thickness. It appears that the only options for the larger LM area categories (3, 4, and 5) would be to cut heavier portions to ensure that steaks met minimum target thicknesses.

**Table 4.** Portioning outcomes for ribeyes stratified by portion weight within LM area category

				Yields^*b*^	
LM area category^*a*^	Portion weight, g	Number of steaks	Average steak thickness, mm	Steak yield, %	Lean trim, %
1	226.8	18.8	21.2	69.2	3.1
	283.5	15.0	26.6	69.0	3.3
	340.2	12.4	31.9	68.5	3.9
	396.9	10.6	37.2	68.0	4.4
2	226.8	20.2	20.6	70.2	2.9
	283.5	16.1	25.8	69.7	3.6
	340.2	13.4	31.1	69.5	3.6
	396.9	11.5	36.2	70.0	3.1
3	226.8	21.8	18.9	70.5	2.7
	283.5	17.3	23.8	70.0	3.1
	340.2	14.3	28.7	69.2	3.9
	396.9	12.3	33.4	69.4	3.7
4	226.8	22.5	18.5	71.3	2.3
	283.5	17.9	23.4	70.8	3.0
	340.2	14.8	28.5	70.4	3.3
	396.9	12.7	32.8	70.2	3.5
5	226.8	25.1	17.4	71.3	2.6
	283.5	19.9	22.0	70.9	3.0
	340.2	16.6	26.5	70.7	3.2
	396.9	14.1	30.8	70.3	3.6

^*a*^LM area categories: 1 = 74.8–80.6 cm^2^; 2 = 81.3–87.1 cm^2^; 3 = 87.7–93.5 cm^2^; 4 = 94.2–100.0 cm^2^; and 5 = 100.6–106.4 cm^2^.

^*b*^Yields of steak and lean trim (face and end cuts not meeting portion weight and/or portion thickness specifications) were estimated using the software from the portioner.

**Table 5.** Portioning outcomes for ribeyes stratified by portion thickness within LM area category

				Yields^*b*^	
LM area category^*a*^	Portion thickness, mm	Number of steaks	Average steak weight, g	Steak yield, %	Lean trim, %
1	19.1	21.2	204.8	70.3	2.1
	25.4	15.9	272.9	70.2	2.2
	31.8	12.6	343.4	69.7	2.6
	38.1	10.4	410.0	69.2	3.2
2	19.1	22.4	208.2	71.2	1.9
	25.4	16.7	278.7	71.3	1.8
	31.8	13.2	348.7	70.7	2.4
	38.1	10.8	423.8	69.8	3.3
3	19.1	22.1	226.0	71.3	1.8
	25.4	16.5	302.8	71.3	1.8
	31.8	13.1	380.9	71.0	2.1
	38.1	10.8	458.5	70.4	2.7
4	19.1	22.7	227.0	72.1	1.6
	25.4	16.9	305.5	72.0	1.7
	31.8	13.3	386.6	71.6	2.1
	38.1	11.1	463.5	71.6	2.1
5	19.1	23.0	249.7	72.1	1.8
	25.4	17.2	333.8	71.9	2.0
	31.8	13.7	418.3	71.7	2.2
	38.1	11.4	500.9	71.4	2.5

^*a*^LM area categories: 1 = 74.8–80.6 cm^2^; 2 = 81.3–87.1 cm^2^; 3 = 87.7–93.5 cm^2^; 4 = 94.2–100.0 cm^2^; and 5 = 100.6–106.4 cm^2^.

^*b*^Yields of steak and lean trim (face and end cuts not meeting portion weight and/or portion thickness specifications) were estimated using the software from the portioner.

For the ribeyes portioned by thickness (Table 5), the question is at what weights steaks will be when the 31.8 mm target thickness is applied. Again, as was observed in the portion weight analysis for ribeyes, LM area categories 1 and 2 produced steaks that were 31.8-mm thick and were around the 340.2 g weight (343.4 and 348.7 g, respectively). Steaks that were 31.8 mm from the LM area categories 3 and 4 were similar in weight (380.9 and 386.6 g, respectively) but would be about 40 g heavier than those in the LM area category 1. As might be expected, steaks that were 31.8-mm thick in LM area category 5 were the heaviest numerically (418.3 g), almost 70 g or 20% heavier than those from the smallest LM area categories. If it is important to have steaks that weigh about 340 g as a target, then ribeyes from LM area category 5 must be portioned into 25.4-mm-thick steaks to do so.

For the strip loins portioned by weight (Table 6), steaks were numerically thicker than was observed for the steaks from the ribeyes, which allowed more flexibility in the number of LM area categories that would qualify to meet target thicknesses. Steaks that met the 31.8-mm thickness target could be achieved as 340.2-g portions for LM area categories 1 and 2, and they approached that target thickness for LM area categories 3 and 4. LM area category 5 produced 340.2-g steaks that were only 29.9-mm thick, but that was at least 3 mm thicker than was found for the same combination for ribeyes. The more rectangular shape of strip loin steaks compared to the more oval-shaped ribeye steaks may provide strip loins a slight thickness advantage when cut into comparable weight portions. This may be especially evident in the strip loin as steaks are cut closer to the caudal versus the cranial end.

**Table 6.** Portioning outcomes for strip loins stratified by portion weight within LM area category

				Yields^*b*^	
LM area category^*a*^	Portion weight, g	Number of steaks	Average thickness, mm	Steak yield, %	Lean trim, %
1	226.8	17.5	22.4	69.1	3.5
	283.5	14.0	28.0	69.0	3.6
	340.2	11.5	33.7	68.3	4.3
	396.9	9.8	39.6	67.5	5.1
2	226.8	17.5	22.2	71.0	3.2
	283.5	13.9	27.6	70.2	3.9
	340.2	11.5	33.4	69.7	4.5
	396.9	9.7	39.0	68.8	5.2
3	226.8	19.6	20.1	72.0	3.1
	283.5	15.6	25.3	71.4	3.7
	340.2	12.9	30.6	70.9	4.2
	396.9	10.9	35.6	70.7	4.5
4	226.8	19.2	20.6	68.7	2.9
	283.5	15.4	25.8	68.9	3.6
	340.2	12.9	31.3	69.1	3.5
	396.9	10.8	36.4	68.0	4.6
5	226.8	20.7	19.6	69.0	2.5
	283.5	17.1	24.6	70.8	3.9
	340.2	14.0	29.9	70.4	4.0
	396.9	11.6	34.6	70.4	4.3

^*a*^LM area categories: 1 = 74.8–80.6 cm^2^; 2 = 81.3–87.1 cm^2^; 3 = 87.7–93.5 cm^2^; 4 = 94.2–100.0 cm^2^; and 5 = 100.6–106.4 cm^2^.

^*b*^Yields of steak and lean trim (face and end cuts not meeting portion weight and/or portion thickness specifications) were estimated using the software from the portioner.

For strip loins portioned by thickness (Table 7), steaks that were 31.8 mm neared but did not exceed 340.2-g portions when sourced from LM area categories 1 and 2. Steaks portioned at 31.8 mm from LM area categories 3 and 4 had steaks that weighed about 30 g more than the targeted 340.2 g, which may give some merchandising flexibility for them because of their proximity to this weight. The 31.8-mm-thick steaks from LM area category 5 produced steaks that approached the 396.9 g weight, which may allow a steak cut this thick to be on the upper range of what may be appropriate for some menus or for some case-ready options. Additionally, if portions that are around the 396.9-g weight range are useful for some foodservice or retail channels, sourcing them from strip loins that are from the LM area categories 1 and 2 would allow thicknesses of up to 38.1 mm to be used, which may provide options for very appealing plate presentations to certain customers/consumers.

**Table 7.** Portioning outcomes for strip loins stratified by portion thickness within LM area category

				Yields^*b*^	
LM area category^*a*^	Portion thickness, mm	Number of steaks	Average steak weight, g	Steak yield, %	Lean trim, %
1	19.1	20.0	201.9	70.3	2.3
	25.4	14.8	270.0	69.8	2.8
	31.8	11.8	337.7	69.2	3.3
	38.1	9.7	404.8	68.5	4.1
2	19.1	20.0	200.8	71.8	2.4
	25.4	14.8	269.9	71.5	2.7
	31.8	11.7	339.0	70.9	3.2
	38.1	9.7	405.3	70.3	3.9
3	19.1	20.3	222.0	72.9	2.2
	25.4	15.1	298.5	72.7	2.4
	31.8	12.0	372.7	72.3	2.8
	38.1	10.0	444.0	71.8	3.4
4	19.1	20.3	219.6	70.3	2.3
	25.4	15.1	292.7	69.8	2.8
	31.8	11.9	369.7	69.2	3.4
	38.1	9.9	441.6	68.7	3.9
5	19.1	21.2	233.1	72.5	2.2
	25.4	15.7	313.3	72.0	2.7
	31.8	12.4	393.4	71.6	3.0
	38.1	10.3	470.6	71.2	3.5

^*a*^LM area categories: 1 = 74.8–80.6 cm^2^; 2 = 81.3–87.1 cm^2^; 3 = 87.7–93.5 cm^2^; 4 = 94.2–100.0 cm^2^; and 5 = 100.6–106.4 cm^2^.

^*b*^Yields of steak and lean trim (face and end cuts not meeting portion weight and/or portion thickness specifications) were estimated using the software from the portioner.

Tenderloins portioned by weight (Table 8) or by thickness (Table 9) allowed more flexibility in creating multiple endpoint outcomes. Because the tenderloin, as a subprimal or as steaks, commands among the highest prices in the marketplace, having more options for weight and thickness combinations increases subprimal utilization across sectors and consumer price points.

**Table 8.** Portioning outcomes for tenderloins stratified by portion weight within LM area category

				Yields^*b*^	
LM area category^*a*^	Portion weight, g	Number of steaks	Average steak thickness, mm	Steak yield, %	Lean trim, %
1	141.8	12.7	34.1	62.1	3.2
	170.1	10.4	39.6	61.2	4.0
	198.5	8.8	45.1	60.6	4.6
	226.8	7.8	51.8	61.4	3.7
	255.2	6.8	57.0	60.2	5.0
	283.5	6.1	62.5	59.2	6.0
2	141.8	13.2	33.6	61.8	3.1
	170.1	10.9	39.0	61.0	3.8
	198.5	9.2	45.0	61.1	3.7
	226.8	8.0	51.6	59.9	5.0
	255.2	6.9	56.4	58.9	6.0
	283.5	6.4	62.8	59.3	5.5
3	141.8	13.8	31.8	62.5	2.9
	170.1	11.4	37.5	61.5	3.9
	198.5	9.1	43.0	61.5	4.0
	226.8	8.4	49.1	60.8	4.6
	255.2	7.4	55.0	59.9	5.6
	283.5	6.6	60.0	59.8	5.6
4	141.8	14.3	31.5	62.4	2.8
	170.1	11.8	36.6	61.8	3.3
	198.5	9.9	42.5	61.7	3.5
	226.8	8.6	48.4	60.0	5.2
	255.2	7.6	53.7	59.6	5.5
	283.5	6.9	58.5	60.3	4.9
5	141.8	14.9	30.4	63.6	2.9
	170.1	12.4	35.5	62.9	3.3
	198.5	10.2	40.6	61.8	4.4
	226.8	9.2	46.5	62.4	3.7
	255.2	8.1	51.9	61.8	4.3
	283.5	7.2	56.3	61.0	5.2

^*a*^LM area categories: 1 = 74.8–80.6 cm^2^; 2 = 81.3–87.1 cm^2^; 3 = 87.7–93.5 cm^2^; 4 = 94.2–100.0 cm^2^; and 5 = 100.6–106.4 cm^2^.

^*b*^Yields of steak and lean trim (face and end cuts not meeting portion weight and/or portion thickness specifications) were estimated using the software from the portioner.

**Table 9.** Portioning outcomes for tenderloins stratified by portion thickness within LM area category

				Yields^*b*^	
LM area category^*a*^	Portion thickness, mm	Number of steaks	Average steak weight, g	Steak yield, %	Lean trim, %
1	25.4	21.6	86.2	64.2	0.9
	31.8	17.0	109.3	64.2	1.0
	38.1	14.1	131.1	64.1	1.1
	44.5	12.0	154.7	64.1	1.0
	50.8	10.4	177.8	64.0	1.2
	57.2	9.1	203.1	63.9	1.3
	63.5	8.1	226.4	63.8	1.4
2	25.4	22.3	86.8	63.9	0.9
	31.8	17.5	110.2	63.8	1.0
	38.1	14.5	133.3	63.8	1.0
	44.5	12.4	155.3	63.7	1.1
	50.8	10.8	177.0	63.8	1.1
	57.2	9.5	202.8	63.6	1.2
	63.5	8.5	227.6	63.5	1.3
3	25.4	21.8	92.6	64.5	0.9
	31.8	17.4	116.1	64.5	0.9
	38.1	14.4	140.2	64.5	1.0
	44.5	12.2	165.4	64.4	1.0
	50.8	10.7	188.9	64.4	1.0
	57.2	9.4	213.0	64.3	1.1
	63.5	8.4	237.8	64.3	1.2
4	25.4	22.3	93.6	64.3	0.9
	31.8	17.7	117.2	64.2	1.0
	38.1	14.7	141.3	64.2	1.0
	44.5	12.5	167.2	64.1	1.1
	50.8	10.9	190.9	64.1	1.1
	57.2	9.6	217.5	63.9	1.3
	63.5	8.5	242.6	63.8	1.4
5	25.4	22.1	98.5	65.3	0.9
	31.8	17.4	124.9	65.2	1.0
	38.1	14.4	151.1	65.1	1.1
	44.5	12.3	176.9	65.1	1.1
	50.8	10.6	203.8	64.9	1.1
	57.2	9.4	230.7	64.9	1.3
	63.5	8.4	257.7	64.8	1.4

^*a*^LM area categories: 1 = 74.8–80.6 cm^2^; 2 = 81.3–87.1 cm^2^; 3 = 87.7–93.5 cm^2^; 4 = 94.2–100.0 cm^2^; and 5 = 100.6–106.4 cm^2^.

^*b*^Yields of steak and lean trim (face and end cuts not meeting portion weight and/or portion thickness specifications) were estimated using the software from the portioner.

The target thicknesses for the tenderloin steaks were 44.5 and 50.8 mm and, when the tenderloin was portioned by weight (Table 8), those in the 198.5 and 226.8 g approached or met the thickness targets for LM area categories 1, 2, and 3. For LM area categories 4 and 5, tenderloin steaks that approached or met the 44.5 and 50.8 mm targets required moving to heavier weights (226.8 and 255.2 g) to satisfy these constraints. None of the tenderloin steaks from any of the LM area categories met the minimum thickness (44.5 mm) for the 170.1 g weight, which is a very popular menu offering for U.S. restaurants. It is clear that sourcing tenderloins from carcasses having LM areas in the range we used would make it difficult to have lighter-weight tenderloin steaks that would meet minimum steak thicknesses.

Because there are two target thicknesses for tenderloin steaks, the combinations of portion thicknesses that approach or meet portion weights provide more options than for the ribeyes and strip loins (Table 9). If tenderloin steaks are cut 50.8-mm thick, then tenderloins from the LM area categories 1 and 2 can weigh approximately 170.1 g. To meet this same approximate weight target, tenderloins from LM area categories 3, 4, and 5 would have to be cut 44.5-mm thick. As might be expected, there are many LM area category/portion thickness combinations that would allow heavier portion weights to be achieved with thicker portions. This is especially evident in those from the LM area categories 1 and 2 where tenderloin steaks could be 57.2- and 63.5-mm thick and would be close to the 198.5 and 226.8 g endpoints—thick portions but not too heavy for most foodservice menu options.

Yields of steaks and lean trimmings for each of the subprimal/portion method (Tables 4–9) revealed two interesting trends. As steaks were portioned with increasing thicknesses or weights, it appears that the yields of lean trimmings numerically increased. Because most of the lean trimmings were the facings or end cuts, it may be that these thicker/heavier portions resulted in slightly greater end cuts than when thinner/lighter portions were generated. The second observation was that steaks portioned by thickness versus weight appeared to have numerically less lean trimmings, especially for the tenderloins (Tables 8 and 9). Both of these trends are worth investigating as manufacturers make merchandising decisions on how steaks are to be portioned.

### Portion Numbers for Each Subprimal

We compared the number of steaks that could be derived from each subprimal using the different portioning methods—by thickness or by weight—to see if LM area influenced steak number (Tables 10–15). For ribeyes (Tables 10 and 11), greater numbers of steaks were seen from larger LM area categories (*P* < 0.0001) and, although this may have been expected, the differences in the number of steaks between the two methods is interesting. As a way to compare the two methods, the difference between the numbers of steaks obtained from LM area category 1 and LM area category 5 was divided by the number in LM area category 1 to calculate a percentage difference. When portioned by weight (Table 10), there were over 34% more steaks by this method, but only about 9% more when portioned by thickness (Table 11). The same trend was found for the strip loins where differences (*P* < 0.0001) occurred for the numbers of steaks from each portion method with about 18% more steaks from the strip loins portioned by weight (Table 12), but only about 6% more when portioned by thickness (Table 13). The LM area categories, of course, were designed to differ by area, not weight or any other measurement, so it may not be surprising that the number of steaks derived from each method may differ. For tenderloin steaks, although there were significant differences in steak numbers for all but one of the portion weight categories (Table 14), there were no (*P* > 0.05) differences found for the number of steaks when portioned by thickness (Table 15). It appears that tenderloins sourced from the different LM area categories probably differed more in their width than in their length. It is interesting to observe how the number of steaks from these subprimals was impacted by the LM area category and by the method of portioning.

**Table 10.** Least squares means (±SEM) for portion number stratified by portion weight within LM area categories for ribeyes

	Portion weight			
LM area category^*a*^	226.8 g	283.5 g	340.2 g	396.9 g
1	18.8^d^ ± 0.4	15.0^c^ ± 0.3	12.4^d^ ± 0.3	10.6^d^ ± 0.2
2	20.2^cd^ ± 0.4	16.1^c^ ± 0.3	13.4^cd^ ± 0.3	11.5^c^ ± 0.2
3	21.8^bc^ ± 0.4	17.3^b^ ± 0.3	14.3^bc^ ± 0.3	12.3^bc^ ± 0.2
4	22.5^b^ ± 0.4	17.9^b^ ± 0.3	14.8^b^ ± 0.3	12.7^b^ ± 0.2
5	25.1^a^ ± 0.4	19.9^a^ ± 0.3	16.6^a^ ± 0.3	14.1^a^ ± 0.2
*P*-value	<0.0001	<0.0001	<0.0001	<0.0001

^*a*^LM area categories: 1 = 74.8–80.6 cm^2^; 2 = 81.3–87.1 cm^2^; 3 = 87.7–93.5 cm^2^; 4 = 94.2–100.0 cm^2^; and 5 = 100.6–106.4 cm^2^.

^a,b,c,d^Means within each column with different superscripts differ (*P* < 0.05).

**Table 11.** Least squares means (±SEM) for portion number stratified by portion thickness within LM area categories for ribeyes

	Portion thickness			
LM area category^*a*^	19.1 mm	25.4 mm	31.8 mm	38.1 mm
1	21.2^b^ ± 0.2	15.9^b^ ± 0.2	12.6^b^ ± 0.2	10.4^c^ ± 0.1
2	22.4^a^ ± 0.2	16.7^a^ ± 0.2	13.2^a^ ± 0.2	10.8^bc^ ± 0.1
3	22.1^a^ ± 0.2	16.5^ab^ ± 0.2	13.1^ab^ ± 0.2	10.8^bc^ ± 0.1
4	22.7^a^ ± 0.2	16.9^a^ ± 0.2	13.3^a^ ± 0.2	11.1^ab^ ± 0.1
5	23.0^a^ ± 0.2	17.2^a^ ± 0.2	13.7^a^ ± 0.2	11.4^a^ ± 0.1
*P*-value	<0.0001	<0.0001	<0.0001	<0.0001

^*a*^LM area categories: 1 = 74.8–80.6 cm^2^; 2 = 81.3–87.1 cm^2^; 3 = 87.7–93.5 cm^2^; 4 = 94.2–100.0 cm^2^; and 5 = 100.6–106.4 cm^2^.

^a,b,c^Means within each column with different superscripts differ (*P* < 0.05).

**Table 12.** Least squares means (±SEM) for portion number stratified by portion weight within LM area categories for strip loins

	Portion weight			
LM area category^*a*^	226.8 g	283.5 g	340.2 g	396.9 g
1	17.5^c^ ± 0.4	14.0^c^ ± 0.3	11.5^c^ ± 0.3	9.8^b^ ± 0.2
2	17.5^c^ ± 0.4	13.9^c^ ± 0.3	11.5^c^ ± 0.3	9.7^b^ ± 0.2
3	19.6^b^ ± 0.4	15.6^b^ ± 0.4	12.9^b^ ± 0.3	10.9^a^ ± 0.2
4	19.2^b^ ± 0.4	15.4^b^ ± 0.3	12.9^b^ ± 0.3	10.8^a^ ± 0.2
5	21.2^a^ ± 0.4	17.1^a^ ± 0.4	14.0^a^ ± 0.3	10.3^a^ ± 0.2
*P*-value	<0.0001	<0.0001	<0.0001	<0.0001

^*a*^LM area categories: 1 = 74.8–80.6 cm^2^; 2 = 81.3–87.1 cm^2^; 3 = 87.7–93.5 cm^2^; 4 = 94.2–100.0 cm^2^; and 5 = 100.6–106.4 cm^2^.

^a,b,c^Means within each column with different superscripts differ (*P* < 0.05).

**Table 13.** Least squares means (±SEM) for portion number stratified by portion thickness within LM area categories for strip loins

	Portion thickness			
LM area category^*a*^	19.1 mm	25.4 mm	31.8 mm	38.1 mm
1	20.0^b^ ± 0.3	14.8^b^ ± 0.2	11.8^b^ ± 0.2	9.7^b^ ± 0.1
2	20.0^b^ ± 0.3	14.8^b^ ± 0.2	11.7^b^ ± 0.2	9.7^b^ ± 0.1
3	20.3^ab^ ± 0.3	15.1^ab^ ± 0.2	12.0^ab^ ± 0.2	10.0^ab^ ± 0.1
4	20.3^ab^ ± 0.3	15.1^ab^ ± 0.2	11.9^ab^ ± 0.2	9.9^ab^ ± 0.1
5	21.2^a^ ± 0.3	15.7^a^ ± 0.2	12.4^a^ ± 0.2	10.3^a^ ± 0.1
*P*-value	0.0073	<0.0001	<0.0001	0.0208

^*a*^LM area categories: 1 = 74.8–80.6 cm^2^; 2 = 81.3–87.1 cm^2^; 3 = 87.7–93.5 cm^2^; 4 = 94.2–100.0 cm^2^; and 5 = 100.6–106.4 cm^2^.

^a,b,c^Means within each column with different superscripts differ (*P* < 0.05).

**Table 14.** Least squares means (±SEM) for portion number stratified by portion weight within LM area categories for tenderloins

	Portion weight					
LM area category^*a*^	141.7 g	170.1 g	198.4 g	226.8 g	255.1 g	283.5 g
1	12.7^c^ ± 0.4	10.4^c^ ± 0.3	8.8 ± 0.3	7.8^b^ ± 0.2	6.8^b^ ± 0.2	6.1^c^ ± 0.2
2	13.2^bc^ ± 0.4	10.9^bc^ ± 0.3	9.2 ± 0.4	8.0^b^ ± 0.2	6.9^b^ ± 0.2	6.4^bc^ ± 0.2
3	13.8^abc^ ± 0.4	11.4^abc^ ± 0.3	9.1 ± 0.3	8.4^ab^ ± 0.2	7.4^ab^ ± 0.2	6.6^abc^ ± 0.2
4	14.3^ab^ ± 0.4	11.8^ab^ ± 0.3	9.9 ± 0.3	8.6^ab^ ± 0.2	7.6^ab^ ± 0.2	6.9^ab^ ± 0.2
5	14.9^a^ ± 0.4	12.4^a^ ± 0.3	10.2 ± 0.3	9.2^a^ ± 0.2	8.1^a^ ± 0.2	7.2^a^ ± 0.2
*P*-value	0.0008	0.0003	0.0596	0.0008	0.0005	0.0011

^*a*^LM area categories: 1 = 74.8–80.6 cm^2^; 2 = 81.3–87.1 cm^2^; 3 = 87.7–93.5 cm^2^; 4 = 94.2–100.0 cm^2^; and 5 = 100.6–106.4 cm^2^.

^a,b,c^Means within each column with different superscripts differ (*P* < 0.05).

**Table 15.** Least squares means (±SEM) for portion number stratified by portion thickness within LM area categories for tenderloins

	Portion thickness						
LM area category^*a*^	25.4 mm	31.8 mm	38.1 mm	44.5 mm	50.8 mm	57.2 mm	63.5 mm
1	21.6 ± 0.3	17.0 ± 0.2	14.1 ± 0.2	12.0 ± 0.2	10.4 ± 0.2	9.1 ± 0.1	8.1 ± 0.1
2	22.3 ± 0.3	17.5 ± 0.2	14.5 ± 0.2	12.4 ± 0.2	10.8 ± 0.2	9.5 ± 0.2	8.5 ± 0.1
3	21.8 ± 0.3	17.4 ± 0.2	14.4 ± 0.2	12.2 ± 0.2	10.7 ± 0.2	9.4 ± 0.1	8.4 ± 0.1
4	22.3 ± 0.3	17.7 ± 0.2	14.7 ± 0.2	12.5 ± 0.2	10.9 ± 0.1	9.6 ± 0.1	8.5 ± 0.1
5	22.1 ± 0.3	17.4 ± 0.2	14.4 ± 0.2	12.3 ± 0.2	10.6 ± 0.1	9.4 ± 0.1	8.4 ± 0.1
*P*-value	0.2397	0.2320	0.1467	0.3817	0.2299	0.1107	0.1735

^*a*^LM area categories: 1 = 74.8–80.6 cm^2^; 2 = 81.3–87.1 cm^2^; 3 = 87.7–93.5 cm^2^; 4 = 94.2–100.0 cm^2^; and 5 = 100.6–106.4 cm^2^.

### Sorting Subprimals by Weight

Under current marketing schemes, there are limited options for retail and foodservice providers to obtain beef subprimals that have been sized in some manner. Beef suppliers may sort some subprimals using the industry jargon terms, “ups” and “downs,” based on weight breaks. USDA (2020c) Agricultural Marketing Service price reporting uses “light” and “heavy” where there are price differences for some products, but there are no published weights listed for these reports. Over time and with the increasing carcass weights the industry has experienced, these weight breaks have increased, so what was an “up” years ago may now be a “down” today.

We asked the beef packer collaborator for guidance about the weight breaks that were most used by its establishments and were given these weights: ribeyes—“downs” = ≤7.71 kg, “ups” = >7.72 kg; strip loins—“downs” = ≤6.80 kg, “ups” = >6.81 kg; and tenderloins—“downs” = ≤3.18 kg, “ups” = >3.18 kg. We then sorted the ribeyes, strip loins, and tenderloins into those weight break categories to determine some of the product characteristics of each (Table 16). For the ribeyes and strip loins, most of the products fell into the lighter-weight categories, whereas, for the tenderloins, there were fewer in the lighter and more in the heavier categories, at least numerically, than were observed for the other two subprimals. The mean LM areas for each subprimal/weight break category look promising as far as sorting LM areas, but what was apparent, when the minimum and maximum LM areas were evaluated, was that there may have been even more variation, especially in the lighter-weight categories for the ribeye and strip loins. Using weight breaks as a proxy for size sorting may eliminate some variation in LM areas for ribeyes and strip loins, but not enough to ensure the uniformity of raw materials that would be of value to retailers and foodservice operators.

**Table 16.** Mean, minimum, and maximum LM areas for ribeyes, strip loins, and tenderloins when sorted by weight breaks

	Subprimal weight breaks^*a*^					
	Ribeyes (*n* = 97)		Strip loins (*n* = 98)		Tenderloins (*n* = 95)	
	≤7.71 kg	>7.72 kg	≤6.80 kg	>6.81 kg	≤3.18 kg	>3.19 kg
Percentage of *n*	84.5	15.5	83.6	16.4	57.9	42.1
Mean LM area, cm^2^	88.5	102.9	88.4	100.5	87.8	96.2
Minimum LM area, cm^2^	75.1	97.2	75.1	90.3	75.1	75.3
Maximum LM area, cm^2^	105.7	106.3	105.7	106.3	106.1	106.2

^*a*^Subprimal weight breaks at boxing based on recommendation from major beef packer.

### Availability of LM Area Categories by Quality Grade/Group

Using the raw data from the NBQA-2016 published by Boykin et al. (2017), we determined the percentage frequencies of each LM area category used for this study by quality grades or groups to see what proportions may be available in the marketplace (Table 17). The observed trends in the percentage frequencies were that the quality grades or groups that have higher marbling scores (e.g., Prime and Premium Choice) had about two-thirds of carcasses in LM area categories 1, 2, and 3, whereas, for lower marbling scores, such as Select, only about half of carcasses were in these categories. Boykin et al. (2017) reported differences (*P* < 0.05) in LM areas among Prime (84.5 cm^2^), Choice (88.9 cm^2^), and Select (91.5 cm^2^), which are reflected somewhat in these percentage distributions. The percentage distribution of carcasses that are in LM area categories 4 and 5 reflect that Select had more than twice as many as Prime and numerically more than either Premium Choice or Choice. It is clear that if packer/processors were to enact an LM area sorting system, carcass numbers would be impacted by the quality grade or group of carcasses used to source them.

**Table 17.** Percentage distribution of carcasses from the LM area categories present in various quality grades/groups from data from the National Beef Quality Audit—2016^*a*^

		LM area category, %^*c*^				
Quality grade/group^*b*^	*n*	1	2	3	4	5
Prime	323	21.7	22.3	21.9	9.6	3.7
Premium Choice	2,522	19.5	21.3	24.8	13.2	7.7
Choice	3,274	14.9	20.2	25.6	15.0	9.9
Select	2,000	13.7	17.1	21.6	17.8	12.1

^*a*^These frequencies were generated using the raw data from Boykin et al. (2017) from the National Beef Quality Audit—2016.

^*b*^ USDA (2017) quality grades. Premium Choice refers to those carcasses that have modest and moderate marbling scores, and Choice are those carcasses that have small marbling. Prime and Select would be the full range of each grade, respectively.

^*c*^LM area categories: 1 = 74.8–80.6 cm^2^; 2 = 81.3–87.1 cm^2^; 3 = 87.7–93.5 cm^2^; 4 = 94.2–100.0 cm^2^; and 5 = 100.6–106.4 cm^2^.

### Challenges With Implementing Sorting by LM Area

Although three different subprimals were sourced for this current work, sorting based on LM area is most effective for the subprimals that actually contain the M. longissimus thoracis (ribeyes) or M. longissimus lumborum (strip loins). Bass et al. (2009) evaluated the suitability of 14 different muscles from carcasses that varied in LM area from 67.7 to 116.1 cm^2^. Of these muscles, variation in the LM area did not affect (*P* > 0.05) the retail portion size or the surface area of the steak cross-sectional face of the psoas major (tenderloin). In the current study, variation in LM area was not as closely associated with portion thickness or portion weights for the tenderloin as it was for the ribeye and strip loin.

There is no clear LM area target that will work for all markets. Sweeter et al. (2005) used ribeyes from carcasses ranging in LM areas in a two-part study: 1) disappearance/steaks sold in an actual retail store setting and 2) willingness-to-pay for steaks from different-sized ribeyes, including a group where steaks had been cut in half. Their work showed no preference for steaks from any of the LM area categories at retail and, for the willingness-to-pay trial, consumers were willing to pay more for steaks from the larger LM area compared to the average LM area, with the steaks cut in half only purchased if at a discount. Cutting large-sized steaks in half has been one of the strategies of the National Cattlemen’s Beef Association Beef Alternative Merchandising (BAM) program (National Cattlemen’s Beef Association, 2009a, 2009b), and programs such as these may become more important as beef subprimals continue to increase in size and weight.

For packer/processors to implement LM area category sortation, there must be consistent demand for such products so that enough production volume could be dedicated to their selection/fabrication/boxing/marketing. One possible method for the selection of carcasses based on LM area would be as a component of a branded beef/certification program. At present, the Certified Angus Beef Program, G-1 specification requirements (USDA, 2019) have minimum (64.5 cm^2^) and maximum (103.2 cm^2^) LM areas, so the mechanism is in place to include such measurements in a program.

With ever-evolving technology to assist in the identification/selection of beef carcass traits and increasing needs for specialized products in the marketplace, the possibility that demand leads to the development of programs that could sort carcasses based on LM area is intriguing. The myriad of combinations of portion thicknesses/portion weights of beef steaks that retail and foodservice need for current and future programs gives hope that innovative ways to source subprimals based on factors, including LM area, will be a great opportunity for the beef industry.
